# Validation of an Open-Source Smartwatch for Continuous Monitoring of Physical Activity and Heart Rate in Adults

**DOI:** 10.3390/s25092926

**Published:** 2025-05-06

**Authors:** Nicholas Ravanelli, KarLee Lefebvre, Amy Brough, Simon Paquette, Wei Lin

**Affiliations:** 1 Heat Resilience & Performance Centre, Yong Loo Lin School of Medicine, National University of Singapore, 27 Medical Drive, #03-01, Singapore 117510, Singapore; 2 Department of Physiology, Yong Loo Lin School of Medicine, National University of Singapore, Singapore 117593, Singapore; 3 Human Potential Translational Research Programme, National University of Singapore, Singapore 117597, Singapore; 4 School of Kinesiology, Lakehead University, Thunder Bay, ON P7B 5E1, Canada

**Keywords:** physical activity, wearables, heart rate, open source, consumer devices, photoplethysmography, validity, agreement

## Abstract

Consumer-grade wrist-based wearable devices have grown in popularity among researchers to continuously collect metrics such as physical activity and heart rate. However, manufacturers rarely disclose the preprocessing sensor data algorithms, and user-generated data are typically shared leading to data governance issues. Open-source technology may address these limitations. This study evaluates the validity of the Bangle.js2 for step counting and heart rate during lab-based validation and agreement with other wearable devices (steps: Fitbit Charge 5; heart rate: Polar H10) in free-living conditions. A custom open-source application was developed to capture the sensor data from the Bangle.js2. Participants (*n* = 47; 25 males; 27 ± 11 years) were asked to complete a lab-based treadmill validation (3 min stages at 2, 3, 4, and 5 mph) and stair climbing procedure followed by a 24 h free-living period. The Bangle.js2 demonstrated systematic undercounting of steps at slower walking speeds with acceptable error achieved at 5 km/h. During free-living conditions, the Bangle.js2 demonstrated strong agreement with the Fitbit Charge 5 for per-minute step counting (CCC = 0.90) and total steps over 24 h (CCC = 0.96). Additionally, the Bangle.js2 demonstrated strong agreement with the Polar H10 for minute-averaged heart rate (CCC = 0.78). In conclusion, the Bangle.js2 is a valid open-source hardware and software solution for researchers interested in step counting and heart rate monitoring in free-living conditions.

## 1. Introduction

The demand for consumer-grade wearable devices has risen dramatically in recent years [1,2]. Increasing numbers of clinical trials are incorporating wearable devices to monitor the health and activity of participants [3]. Unfortunately, researchers are rarely aware of, or granted access to review, the underlying algorithms which preprocess the raw sensor data of wearable devices into key endpoints such as steps, sleep duration and quality, or heart rate [4]. Moreover, software updates within the lifespan of a device could impact study replication, or even risk introducing systematic error within an ongoing longitudinal study [5]. Lastly, the hardware and algorithms used are often lost, or rendered obsolete, following the devices’ end of life.

Data governance has become an increasingly important concern for consumers and researchers who explore the use of wearable devices and activity trackers for clinical trials [6,7]. Many commercially available fitness trackers require synchronization with proprietary software, often hosted online, for data processing without any alternative data access solution. This sharing of user-generated data with the device manufacturer creates an ethical issue for researchers with respect to data governance whereby researchers must agree that participant data will be shared with the manufacturer of the device, and it is the researcher’s responsibility to inform the participant during the consent process. Distributing data to multiple parties increases the risk of a potential data breach, and larger wearable companies are not immune to this reality. For example, 61 million records were recently exposed online from Apple and Fitbit due to a security breach [8]. Furthermore, the consumer, the researcher, or the participant may not have control over the user-generated data shared with the manufacturer, including data that might not be collected for the original research question, which may be distributed with other third-party agents, or used for financial gain at the discretion of the manufacturer [7].

An alternative solution to increase transparency of data processing and enhance data management may be through increasing the adoption of open-source hardware and software alternatives. Open-source technology may also improve repeatability of studies using documented hardware and software for researchers to review. Additionally, access to how data are processed and stored can facilitate maintenance of complete, or near complete, data governance for researchers, which includes how data are collected, produced, utilized, controlled, and reported [9]. Specifically, the open-source activity tracking smartwatch, Bangle.js2 (from Pur3 Ltd., Oxford, UK), permits access to the underlying preprocessing of raw sensor data and stores all data locally on the device in non-volatile memory. The fully offline model provided by the Bangle.js2 provides a unique opportunity for researchers to collect pertinent data while not requiring synchronization with third-party service providers, enhancing data governance and protection of user-generated data akin to lab-based research. However, it remains unknown whether the Bangle.js2, its onboard hardware, and its current firmware is valid and demonstrates good agreement for monitoring per-minute step counts and minute-averaged heart rate, key variables of interest when conducting field-based research.

Thus, the purpose of the present study was to evaluate the validity and agreement of the Bangle.js2 step-counting algorithm against another wrist-based activity tracker (Fitbit Charge 5) during free-living conditions over 24 h and during an in-lab treadmill and stair-climbing assessment. A secondary purpose was to assess the agreement over 24 h of free-living conditions between the Bangle.js2 photoplethysmography sensor for measuring heart rate against a chest-worn electrocardiogram-based heart rate monitor (Polar H10). It was hypothesized that the Bangle.js2 would be valid and demonstrate acceptable agreement for step counting and heart-rate monitoring.

## 2. Methods

### 2.1. Ethical Approval and Recruitment

Ethical approval was obtained from the Lakehead University Research Ethics Board (#1469329) and this study was conducted in accordance with the Declaration of Helsinki, except for registration in a database. In line with the recent consensus statement, a minimum of 45 participants were required to answer the primary objective [10].

### 2.2. Procedures

Two consumer-based wrist-mounted brands (Fitbit Charge 5 and Bangle.js2) were worn on the participants’ non-dominant wrist and the position (e.g., proximal vs. distal) on the wrist was randomized between devices. The accelerometer sampling rates were 12.5 Hz and 100 Hz for the Bangle.js2 and Fitbit Charge 5, respectively. All devices were attached to the person prior to the in-lab assessment and were then returned to the researcher team 24 h after. Participants were instructed to remove all trackers during bathing or prior to swimming and document the times on a log sheet provided by the researchers. The lab-based trial consisted of a stepwise intensity protocol on a treadmill (Woodway) at 4 incremental speeds (2, 3, 4, and 5 mph) with a 0% incline for 3 min at each speed. Manual step counting was conducted in real time by SP for each speed using a hand tally counter. Between each speed level, participants straddled the treadmill belt for ~30 s while the steps registered by each respective watch were recorded by the researcher (SP). An approximate 30 s delay was needed to ensure the device had appropriately updated their step-counting records and refreshed on screen. Next, participants completed 10 circuits of a 9-step staircase located within Sanders Fieldhouse. The number of steps taken for each speed and stair-climbing exercise were counted in real time by the assessor for all sessions (SP). Participants continued to wear the devices for 24 h and returned them to the research team the following day. During data collection for the primary objective (step counting), a firmware update on the Bangle.js2 (V2.19) substituted the low-pass finite impulse response filter for heart-beat detection which demonstrated poor agreement in pilot testing with the proprietary algorithm designed by Vcare (Chengdu, China), the manufacturer of the onboard photoplethysmography sensor (VC31b). We therefore continued recruiting participants to complete only the 24 h free-living condition where we instrumented them with an additional electrocardiogram-based heart-rate strap (Polar H10, sampling rate of 1000 Hz in RR mode) on the xiphisterna line which transmitted data directly to the Bangle.js2 via the Bluetooth communication protocol. The Polar H10 is as accurate as the gold-standard electrocardiogram Holter device for measuring heart rate [11]. The photoplethysmography sensor sampling frequency for the Bangle.js2 and the Fitbit Charge 5 were 25 Hz and 100 Hz, respectively. Participants outfitted with heart-rate monitoring were also asked to self-report their skin type and tone based on the Fitzpatrick Scale [12] and June Robinson Scale [13], respectively. All raw data were immediately downloaded from Bangle.js2 upon return, and the Fitbit Charge 5 was synchronized with Google’s Online Cloud storage server through the Fitbit App. A custom python script for aggregating per-minute step count and minute heart-rate data was used to download the data. The Bangle.js2 watch software, which is part of the HeatSuite platform [14], is open source and available on Github through the Bangle.js App Loader [15], and the python script for downloading data from the Fitbit webserver is publicly available on the Open Science Framework [16].

### 2.3. Data and Statistical Analysis

Non-wear periods were excluded from the statistical analysis (e.g., charging, bathing) and a valid day of data was defined as 16 h or more in the 24 h period. Data were time aligned between the Bangle.js2 and the Fitbit Charge 5 to the nearest whole minute. Minute-averaged heart rate from the Bangle.js2 optical sensor and Polar H10 heart-rate strap were recorded simultaneously to the internal memory of the Bangle.js2.

The agreement between the Bangle.js2 and the Fitbit Charge 5 for step tracking was assessed within devices during the lab trials in comparison to the true steps observed by the researcher, and between devices over 24 h by comparing (i) minute-by-minute data; and (ii) the total steps taken over each valid day. For heart rate, agreement of either the Bangle.js2 or the Fitbit Charge 5 optical sensor to the Polar H10 chest strap was compared at a per-minute resolution. As previous work has demonstrated that movement can impact an optical sensor’s accuracy in measuring heart rate [17,18], a secondary analysis was conducted to determine the agreement between the Bangle.js2 optical sensor or the Fitbit Charge 5 and the Polar H10 heart-rate strap for minute-average heart rate where (i) no steps were detected, or (ii) one or more steps were detected from the Bangle.js2.

Primary agreement analysis included concordance correlation coefficient (CCC), mean absolute error (MAE), mean absolute percent error (MAPE), median absolute percent error (MdAPE for skewed data), typical error (TE), and Bland–Altman analysis. Additionally, a log-transformed Mann–Whitney U analysis was used to compare the similarities in minute-by-minute data distribution during 24 h free-living condition for step count (Bangle.js2 vs. Fitbit Charge 5), and heart rate (Bangle.js2 or Fitbit Charge 5 vs. Polar H10). Recognizing that there would be a large sample size of per-minute step and heart-rate data which may increase the likelihood of statistical significance, 1000 permutations of a subsample were generated at random (*n* = 5000). A Cliff’s Delta was used to assess the effect size for all Mann–Whitney U comparisons and interpreted as negligible (<0.147), small (≥0.147), medium (≥0.33), or large (≥0.474) [19]. All agreement analysis was computed using the python library SciPy, and data visualization was conducted with matplotlib, with original code freely available [16]. All CCC were interpreted using ranges defined in prior wearable research [20]; weak: <0.50, moderate: 0.50 ≤ 0.70, strong 0.70 ≤ 0.99, and almost perfect: >0.99. The acceptable error threshold for MAPE and/or MdAPE was set to ≤10% according to the Consumer Technology Association (CTA) standard for treadmill-based validation [21]. It should be acknowledged that this is not-location specific, and more recent work has proposed acceptable error thresholds for step tracking at the wrist of ≤11% [22], with a higher acceptable error threshold for free-living conditions (≤15%) [10]. Initial pilot testing demonstrated that the FitBit Charge 5 had low measurement error (<5%) for step tracking when assessed using the gold-standard method (manual step counting), thus it was used as the reference device to compare the Bangle.js2 against for free-living step counting. MAPE was omitted for per-minute step-tracking agreement analysis due to the inherent limitations when a large proportion of the true value will likely be 0 (e.g., sleeping or sedentary time). With respect to heart-rate analysis, the acceptable error threshold for MAPE and/or MdAPE was set at ≤10% as recommended by the CTA Standard for Physical Activity Monitoring for Heart Rate in the real world [23]. Bland–Altman plots were used to visualize the bias and trends within and between devices for measuring steps (Bangle.js2 vs. Fitbit Charge 5), or heart rate (Bangle.js2 or Fitbit Charge 5 vs. Polar H10).

## 3. Results

This study recruited a total of 47 participants and their characteristics are presented in Table 1. Of the sample, 23 completed the in-lab session, and 26 completed the 24 h free-living condition while wearing the Polar H10 in addition to the two wrist-based wearables. All individuals completed the 24 h free-living condition, with two participants completing the 24 h free-living condition twice as they participated before and after the firmware update which integrated the proprietary heart-rate algorithm of the photoplethysmography sensor on the Bangle.js2, resulting in a total of 49 independent 24 h observations for step tracking.

### 3.1. In-Person Data Collection

**Figure 1:**
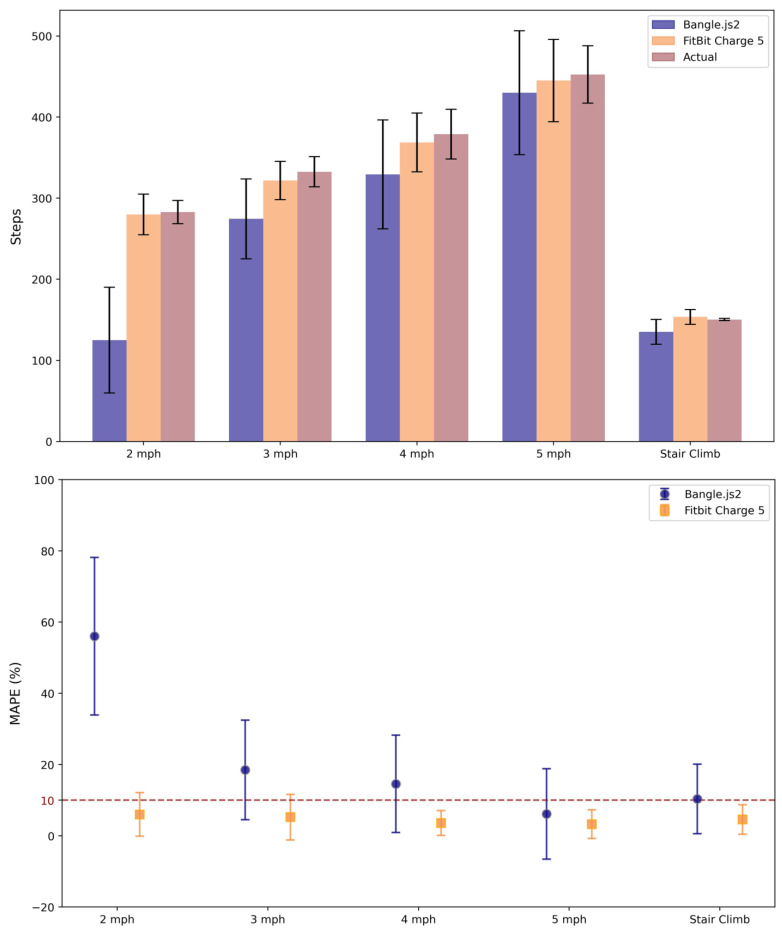
Mean and standard deviation of actual steps observed, and steps counted by the Bangle.js2 and the Fitbit Charge 5 during the treadmill protocol and stair-climb exercise (**top**) with mean absolute percent error (MAPE, **bottom**). The dotted red line denotes the acceptable error threshold defined by the Consumer Technology Association [21].

Figure 1 illustrates the actual and registered step counts and MAPE for each wrist-based wearable during the treadmill-based validation and stair-climbing exercise, and Table 2 reports all outcomes of our agreement analysis. The Bangle.js2 consistently underreported total steps at each speed assessed. MAPE progressively reduced with each increase in speed reaching an acceptable error threshold at 5 mph (6.1%) and the highest error at 2 mph (56.0%). The MAPE of the Fitbit Charge 5 was acceptable at all speeds assessed (mean of all speeds: 4.5 ± 1.1%, range: 3.6–6.0%). The Fitbit Charge 5 was within the acceptable error range for the stair-climbing exercise (4.6%) whereas the Bangle.js2 was near the acceptable error threshold (10.3%). The Bangle.js2 CCC for all walking speeds demonstrated a moderate agreement (0.58 [0.46,0.68]), and a strong agreement was observed in the Fitbit Charge 5 (0.94 [0.93,0.95]).

### 3.2. Twenty-Four-Hour Free-Living Steps

**Figure 2:**
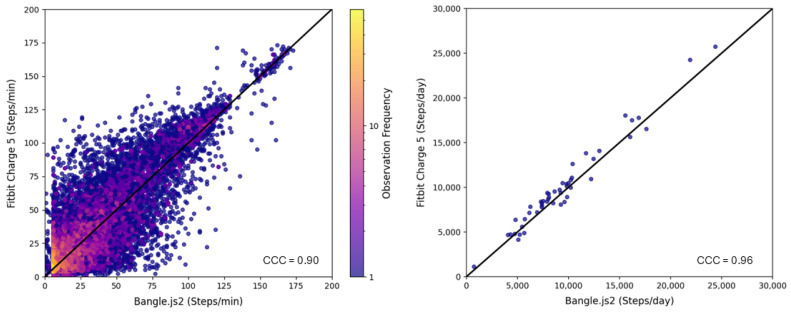
Scatter plot (with line of identity) for the time-aligned relationship of per-minute step counts (**left**) and total steps over a valid day (**right**) between the Bangle.js2 and the Fitbit Charge 5. **CCC:** concordance correlation coefficient.

**Figure 3:**
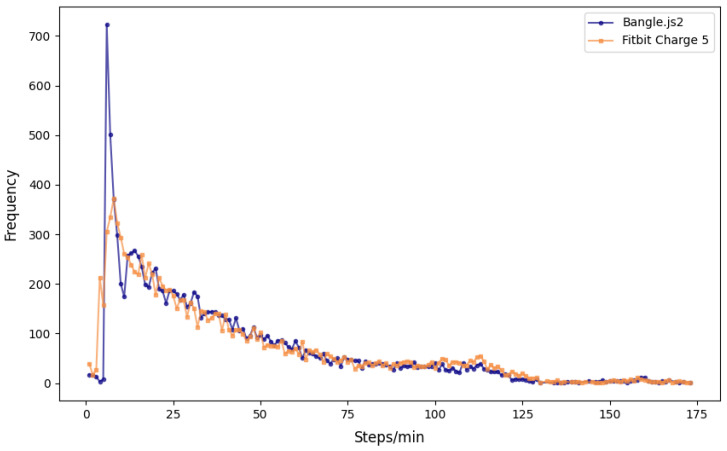
Frequency distribution of the per-minute step counts registered from the Bangle.js2 and the Fitbit Charge 5.

**Figure 4:**
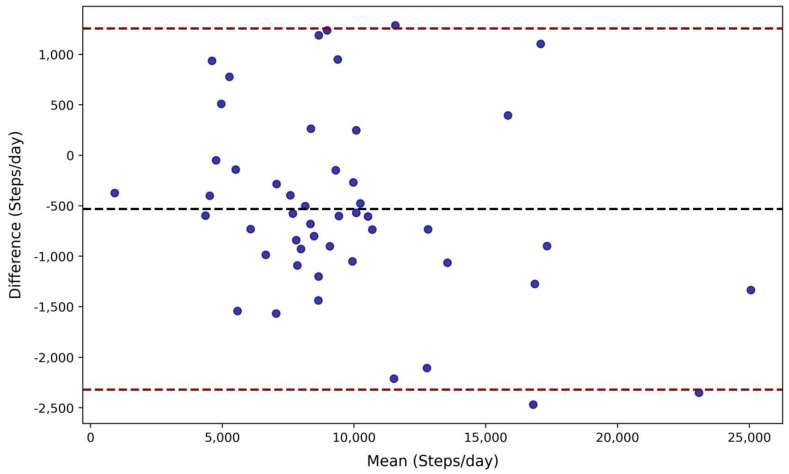
Bland–Altman analysis for total steps recorded per day (Bangle.js2–Fitbit Charge 5). Mean bias and limits of agreement are highlighted by the dashed black and red lines, respectively.

A total of 68,873 independent per-minute observations were made for free-living step tracking between the two wrist-based wearables with an average of 1406 ± 84 per valid ~24 h period (range: 980–1475 min). Figure 2 visualizes the step-counting data per minute and the total sum for each valid day. Table 3 reports the agreement analysis results for free-living step counting. The Mann–Whitney U analysis confirmed the distributions of per-minute step counts were similar between Bangle.js2 and Fitbit Charge 5 (*p* > 0.85), with a Cliff’s Delta inferring negligible difference (−0.014, Figure 3). A strong agreement was observed between the Bangle.js2 and the Fitbit Charge 5 for per-minute data (CCC = 0.90) and total steps counted over 24 h (CCC = 0.96). The total of steps over 24 h with the Bangle.js2 relative to the Fitbit Charge 5 was within acceptable error (<15%, Table 3). Bland–Altman analysis revealed a negative mean bias with the Bangle.js2 relative to the Fitbit Charge 5 suggesting systematic underestimation of total step counts over 24 h (Table 3, Figure 4).

### 3.3. Heart Rate

**Figure 5:**
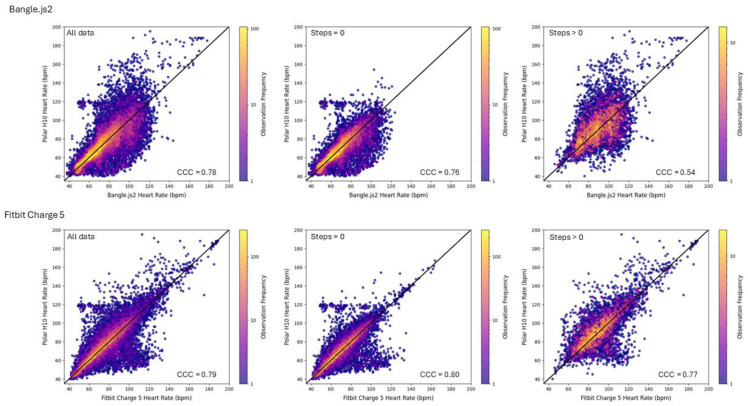
Scatter plots (with line of identity) visualizing the relationship between minute-averaged heart rate reported by the photoplethysmography sensor of the Bangle.js2 (**top** row) or the Fitbit Charge 5 (**bottom** row) compared to the electrocardiogram-based chest strap (Polar H10). Plots are subdivided into (i) all data (**left**); (ii) sedentary (steps = 0, **middle**); or (iii) active (steps > 0, **right**). CCC: concordance correlation coefficient.

**Figure 6:**
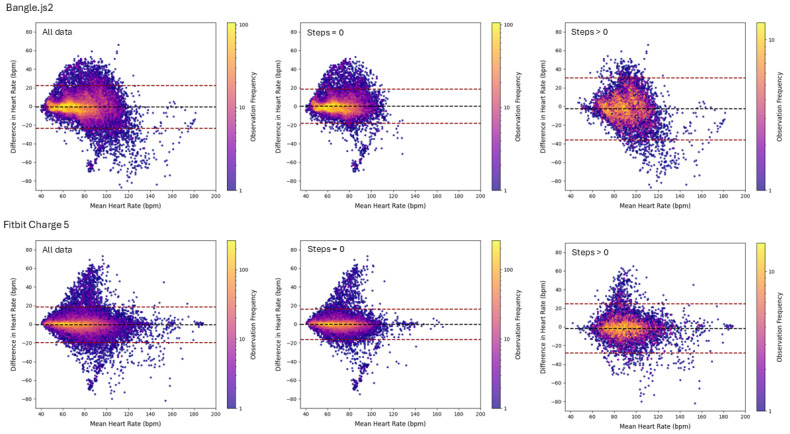
Bland–Altman analysis for minute-averaged heart rate recorded by the photoplethysmography sensor of the Bangle.js2 (**top** row) or the Fitbit Charge 5 (**bottom** row) compared to the electrocardiogram-based chest strap (Polar H10). Plots are subdivided into (i) all data (**left**); (ii) sedentary (steps = 0, **middle**); or (iii) active (steps > 0, **right**).

**Figure 7:**
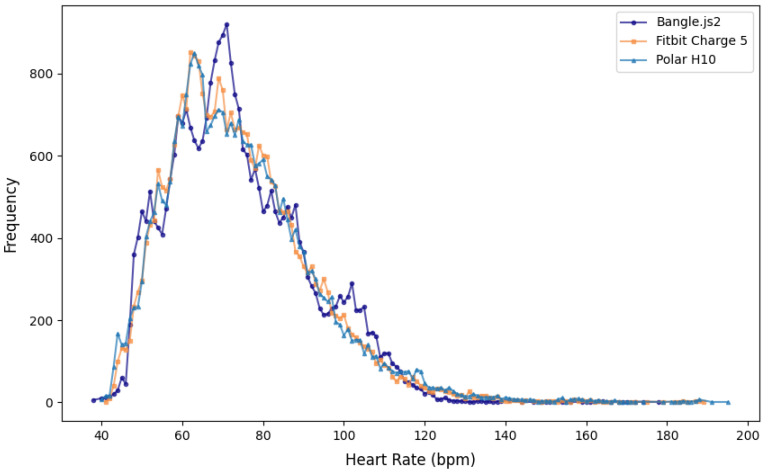
Frequency distribution of the minute-averaged heart rate reported from the Bangle.js2, the Fitbit Charge 5, and the Polar H10 for all free-living assessments.

Table 4 reports all outcomes of the agreement analysis between either the Bangle.js2 or the Fitbit Charge 5 in comparison to the Polar H10. Figure 5 visualizes the relationship between the minute-averaged heart rate for each of the wrist-based devices plotted against the Polar H10, and Figure 6 depicts the results of the Bland–Altman analysis. Mann–Whitney U tests confirmed that the distribution of heart rate reported from the chest-based and wrist-worn heart-rate monitors were similar (*p* > 0.15, Figure 7). The Cliff’s Delta for all comparisons demonstrated negligible effect, confirming the per-minute heart-rate observations, although skewed, were similarly distributed between either group. The Bangle.js2 and the Fitbit Charge 5 demonstrated strong agreement for all observations relative to the Polar H10, and each presented acceptable error (MdAPE < 10%). When evaluating heart-rate measurements during sedentary times (e.g., steps = 0), both wrist-worn wearables demonstrated strong agreement with acceptable error. The Fitbit Charge 5 demonstrated acceptable error and a strong agreement during movement (e.g., steps > 0), whereas the Bangle.js2 retained acceptable error but demonstrated good agreement. For each agreement analysis, the mean absolute error was lower with the Fitbit Charge 5 in comparison to the Bangle.js2. Bland–Altman analysis revealed no mean bias for all data and sedentary periods (mean bias ~0 BPM); however, mean bias was lower during activity periods for the Bangle.js2 (mean bias = −2.4 BPM) and the Fitbit Charge 5 (mean bias = −1.4 BPM).

## 4. Discussion

This study provides the first agreement analysis of the Bangle.js2, an open-source user-programmable smartwatch, for step tracking and heart rate monitoring in adults during in-lab and 24 h free-living conditions. In comparison to actual steps counted during the in-lab validation component, the Bangle.js2 systematically underreported the total steps taken for each speed assessed. The Bangle.js2 was within the acceptable error threshold during the fastest speed tested (5 mph) and was near the acceptable error range during the stair-climbing task (10.3%). The Bangle.js2 demonstrated good agreement for all speeds combined. With respect to the 24 h free-living conditions, the Bangle.js2 showed strong agreement with the Fitbit Charge 5 for per-minute step counting and total over 24 h, with acceptable error. Lastly, heart rate monitoring with the Bangle.js2 demonstrated good agreement against the Polar H10 for all observations, although secondary analysis showed agreement reduced during motion (e.g., steps > 0). Collectively, the present findings suggest that the Bangle.js2 may be a satisfactory open-source solution for continuously monitoring free-living activity and heart rate of healthy adults in free-living conditions.

The most notable observation during the in-lab validation of the treadmill-based step tracking was the systematically lower step counts for each speed with the Bangle.js2, although MAPE progressively improved with increasing speed. Undercounting steps at slower speed is a problem that affects many step counters on the market [24]. For example, Mora-Gonzalez et al. [25] observed a similar trend among wrist, ankle, thigh, and waist activity trackers. In contrast, although a similar progressive improvement in MAPE was observed, the Fitbit Charge 5 demonstrated within acceptable error for all speeds. Due to the closed nature of the underlying software, it is difficult to ascertain the exact reasons for the improved step tracking within the Fitbit Charge 5 algorithm. Fortunately, the open-source nature of the Espruino firmware permits reviewing the underlying code to reveal potential limitations that may be augmented in future iterations to improve accuracy. In brief, the current step-tracking algorithm utilizes a finite state machine for detecting no stepping, the onset of steps, and continuous stepping. Future work could consider modifying the step detection acceleration thresholds, the step timing constraints, the minimum number of steps required to flag registration of steps, and/or assess whether there is over-filtering of small movements which may be true, unregistered, steps. To highlight potential sources of error in the current step-counting algorithm, less than 0.1% of all per-minute step tracking with the Bangle.js2 was between 1–6 steps (Figure 3), which coincides with the firmware gating of ≥6 steps within a specific time domain before registration begins. This likely explains the reduced accuracy in step counting observed during the treadmill protocol, particularly with slower walking speeds. Moreover, Small et al. [26] demonstrated that reducing an accelerometer sampling rate (e.g., from 100 Hz to 25 Hz) impacts activity detection with consistently lower activity being observed. Further refinement of the onboard step detection algorithm should therefore consider increasing the accelerometer sampling rate.

There remains the inherent challenge of maintaining exact time synchronization between devices for conducting agreement analysis during the 24 h free-living period, resulting in potential increased variability in the agreement between per-minute step counting as can be visualized in Figure 2. To account for this potential time domain issue, we further analyzed the frequency distribution of per-minute step counting between both devices and observed a negligible difference. Furthermore, we observed an acceptable error for step counting over the 24 h period with the Bangle.js2 during free-living conditions among the adult population assessed, suggesting it can be a suitable open-source alternative to the Fitbit Charge 5 in its current form. Future work is required to assess the validity of the Bangle.js2 for step counting in populations with slower gait speeds or poor mobility.

The Bangle.js2 includes a low-cost photoplethysmography sensor (Vcare VC31B) for continuously monitoring heart rate; however, only a recent firmware update (+V2.19) enabled use of the proprietary algorithm designed by the sensor manufacturer. Overall, the Bangle.js2 and Fitbit Charge 5 demonstrated strong agreement with acceptable error against the Polar H10 for minute-average heart rate. Furthermore, the frequency of minute-averaged heart rate observations between all three devices demonstrated similar distributions, although the Bangle.js2 had the lowest maximum heart rate recorded (178 BPM) in comparison to the Fitbit Charge 5 (189 BPM) and the reference Polar H10 (195 BPM). Notably, motion can cause greater variability in heart rate measured by wrist-worn devices [17], and thus we conducted a secondary analysis of heart-rate agreement during assumed sedentary (steps = 0) and active periods (steps > 0). The Bangle.js2 retained strong agreement with Polar H10 during sedentary periods with acceptable error, and the agreement reduced to good during activity periods (see Table 4). In contrast, the Fitbit Charge 5 demonstrated strong agreement with the Polar H10, irrespective of activity. Again, the limits of agreement with the Bland–Altman analysis are similar to previous observations at rest and activity from various other wrist-based photoplethysmography sensor heart-rate monitors [18]. Specifically, other low-cost wrist-based heart-rate monitoring devices (e.g., Xiaomi Mi Band) have demonstrated comparable reductions in agreement with increasing activity levels [27]. To our knowledge, this is the first study examining the accuracy of the Vcare VC31B photoplethysmography sensor and its proprietary algorithm. Albeit speculative, the reduced agreement during active periods suggests that Vcare’s proprietary algorithm for handling motion artifacts needs improvement; however, it demonstrated superior agreement in comparison to the finite impulse response filter used in the Espruino firmware for detecting heart beats from the raw photoplethysmography signal (pilot data, unpublished). In support of transparency and reproducibility, future work should aim to improve the pre-existing open-source heart-beat detection algorithm. Alternative filtering techniques of the raw photoplethysmography signal such as the Butterworth or Chebyshev II [28], and/or incorporating detection and removal of motion artifacts using the onboard accelerometer [29], warrant future consideration and validation.

An advantage of open-source software and hardware, like Espruino and the Bangle.js2, is expandability. One can incorporate a more accurate electrocardiogram-based solution for monitoring heart rate like the Polar H10 if needed. Future firmware upgrades may enable additional physiological monitoring potential, such as blood oxygen saturation, which are metrics provided from the Vcare VC31B proprietary algorithm, although validation is required. Additionally, the Bangle.js2 can be adapted for other unique use cases. For example, the Bangle.js was previously used in a custom smartphone application for a low-cost ambulation monitoring in individuals with comorbidities [30]. Furthermore, we have recently integrated the Bangle.js2 into our highly scalable and modular remote health data collection platform, HeatSuite, which enables real-time monitoring of physiological, behavioral, and perceptual responses of end-users to personal environments [14]. However, the development of these or additional features requires technical expertise which may be limiting to some.

Our study is not without limitations. We acknowledge the systematic undercounting of steps at slower walking speeds which warrants further evaluation of its use for step tracking in individuals with slower walking speeds. Previous work has demonstrated the onboard accelerometer of the Bangle.js2 performs comparably to the research-grade ActiGraph GT9X [30], further supporting that refinements to the step detection algorithm are needed. In substitution to step counting, raw accelerometry data may glean insights into physical activity patterns [31], or be used for human activity recognition [32,33]. Although we collected over 30,000 min-averaged heart rate samples, we may be underpowered in our agreement analysis between the Bangle.js2 and the Polar H10 as per recent consensus statements suggesting a minimum of 45 participants [34], warranting future work. Next, there is the risk of potential racial bias in transmittance photoplethysmography analysis whereby darker skin tones align with poorer accuracy [35]. While we were inclusive of all skin tones and types, the maximum self-reported skin type and tone were 3 and 4, respectively. Thus, more work is needed to confirm whether the photoplethysmography sensor demonstrates similar accuracy across different skin types and tones.

## 5. Conclusions

The Bangle.js2 demonstrates moderate agreement for step counting using the treadmill-based validation protocol with error exceeding the acceptable threshold at slower speeds, and strong agreement for free-living monitoring in comparison to another wrist-based alternative (Fitbit Charge 5). The Bangle.js2 demonstrated strong agreement for monitoring minute-averaged heart rate during free-living conditions. Secondary analysis revealed that the strong agreement was largely attributed to the strong agreement observed during sedentary periods, with good agreement observed during activity periods. Collectively, the Bangle.js2 may be a valid low-cost substitute to other commercially available wrist-based wearables for researchers interested in monitoring steps and heart rate among participants in free-living conditions.

## Figures and Tables

**Table 1:** Participant characteristics for each of the three study arms for evaluating agreement of the Bangle.js2 step counting and heart-rate monitoring.

	Lab Validation (*n* = 23)	24 h Step Counting (*n* = 47)	24 h Heart Rate (*n* = 26)
Male/Female	15/8	25/22	12/14
Age (y)	23.0 ± 3.6	26.7 ± 11.3	30.1 ± 14.2
Height (cm)	178 ± 10	176 ± 9	174 ± 9
Weight (kg)	80.6 ± 14.1	78.0 ± 12.9	75.9 ± 11.0
BMI	25.5 ± 4.4	25.2 ± 3.7	24.9 ± 2.8
Fitzpatrick Skin Type	-	-	2.8 ± 0.9
June Robinson Skin Tone	-	-	1.5 ± 0.7

**Table 2:** Agreement analysis results for the Bangle.js2 and Fitbit Charge 5 during 3 min treadmill walking intervals at 4 different speeds, and a stair-climbing exercise.

Condition	Device	MAE [95% CI]	MAPE [95% CI]	MdAPE [95% CI]	TE [95% CI]
2 mph	Bangle.js2	158 [134, 182]	56.0 [47.4, 64.7]	56.5 [35.9, 71.1]	169 [145, 194]
	Fitbit Charge 5	17 [10, 24]	6.0 [3.6, 8.4]	4.8 [2.4, 6.4]	24 [15, 33]
3 mph	Bangle.js2	62 [43, 82]	14.3 [13.0, 24.0]	14.3 [0.3, 23.3]	79 [58, 100]
	Fitbit Charge 5	18 [9, 27]	5.2 [2.7, 7.7]	3.1 [1.2, 4.3]	29 [18, 39]
4 mph	Bangle.js2	55 [35, 75]	14.6 [9.2, 19.9]	11.0 [4.9, 17.4]	75 [53, 97]
	Fitbit Charge 5	13 [8, 19]	3.6 [2.2, 4.9]	2.1 [1.4, 4.0]	19 [13, 25]
5 mph	Bangle.js2	25 [6, 45]	6.1 [1.2, 11.1]	0.8 [0.7, 2.0]	56 [36, 75]
	Fitbit Charge 5	14 [8, 20]	3.3 [1.7, 4.8]	2.3 [1.2, 3.1]	21 [13, 28]
Stair Climb	Bangle.js2	16 [10, 21]	10.3 [6.5, 14.2]	6.7 [4.7, 12.0]	21 [15, 27]
	Fitbit Charge 5	7 [4, 9]	4.6 [2.9, 6.2]	3.3 [1.3, 6.7]	9 [6, 13]

MAE: mean absolute error; MAPE: mean absolute percent error; MdAPE: median absolute percent error; TE: typical error; CI: confidence interval.

**Table 3:** Agreement analysis results for the Bangle.js2 compared against the Fitbit Charge 5 for per-minute step counting and total steps over 24 h of free-living conditions.

	Sample (*n*)	CCC [95% CI]	MAE [95% CI]	MAPE [95% CI]	MdAPE [95% CI]	TE [95% CI]	Bland–Altman Bias [LoA]	Cliff’s Delta
Minute-by-minute steps	68,873	0.90 [0.90, 0.90]	3.5 [3.5,3.6]	-	-	9.3 [9.3, 9.4]	0.2 [−18.1, 18.5]	−0.014
Total 24 h steps	49	0.96 [0.94, 0.96]	1025.7 [810.2, 1241.2]	11.0 [8.5, 13.5]	8.0 [−11.4, 31.7]	1282.3 [11.9, 12.1]	−531 [−2320, 1257]	-

CCC: concordance correlation coefficient; MAE: mean absolute error; MAPE: mean absolute percent error; MdAPE: median absolute percent error; TE: typical error; CI: confidence interval.

**Table 4:** Agreement analysis results for the Bangle.js2 compared against the Polar H10 for minute-averaged heart-rate monitoring during the 24 h free-living conditions.

	Sample (*n*)	CCC [95% CI]	MAE [95% CI]	MAPE [95% CI]	MdAPE [95%CI]	TE [95% CI]	Bland–Altman Bias [LoA]	Cliff’s Delta
All observations (vs. Polar H10)								
Bangle.js2	30,598	0.78 [0.78, 0.78]	7.2 [7.1, 7.3]	9.3 [9.2, 9.4]	5.7 [5.5, 5.7]	11.8 [11.7, 11.9]	−0.3 [−23.2, 22.5]	−0.006
Fitbit Charge 5	30,549	0.79 [0.79, 0.79]	5.4 [5.3, 5.5]	6.9 [6.8, 7.1]	2.8 [2.8, 3.0]	12.0 [11.9, 12.1]	−0.3 [−19.5, 18.8]	0.010
Sedentary (e.g., 0 Steps, vs. Polar H10)								
Bangle.js2	23,322	0.76 [0.76, 0.77]	5.6 [5.5, 5.7]	8.1 [7.9, 8.2]	4.9 [4.8, 5.0]	9.5 [9.4, 9.6]	−0.3 [−18.0, 18.6]	−0.013
Fitbit Charge 5	23,181	0.80 [0.80, 0.80]	3.9 [3.8, 4.0]	5.6 [5.4, 5.7]	2.2 [2.1, 2.2]	8.6 [8.5, 8.7]	−0.0 [−16.5, 16.4]	−0.007
Active (e.g., >0 Steps, vs. Polar H10)								
Bangle.js2	7276	0.54 [0.53, 0.56]	12.5 [12.2, 12.7]	13.2 [12.9, 13.5]	9.9 [9.7, 10.3]	17.2 [16.8, 17.6]	−2.4 [−35.7, 30.8]	0.039
Fitbit Charge 5	7242	0.77 [0.76, 0.78]	8.6 [8.4, 8.9]	9.7 [9.4, 9.9]	6.1 [5.8, 6.2]	13.0 [12.7, 13.3]	−1.4 [−25.0, 27.7]	0.052

CCC: concordance correlation coefficient; MAE: mean absolute error; MAPE: mean absolute percent error; MdAPE: median absolute percent error; TE: typical error; CI: confidence interval.

## Data Availability

The dataset used for the present project, and the code for statistical analysis, graph generation, and aggregating Fitbit data from their webserver, are freely available on the Open Science Framework [16]. The HeatSuite watch application is freely available on the Espruino Bangle.js App Loader [15].
